# Amyotrophic lateral sclerosis and degenerative cervical myelopathy: phenotype-based diagnostic pitfalls, investigative mismatch, and practical clinical reasoning

**DOI:** 10.3389/fneur.2026.1901811

**Published:** 2026-08-06

**Authors:** Dongxia Chen, Tao Shen, Hang Ren, Peng Qiu

**Affiliations:** 1 Department of Encephalopathy/Neurology, Shenzhen Pingle Orthopedic Hospital (Shenzhen Pingshan Traditional Chinese Medicine Hospital), Shenzhen, China; 2 Department of Orthopedic Surgery, Lanzhou University Second Hospital, Lanzhou, China; 3 Department of Spine Surgery, Shenzhen Pingle Orthopedic Hospital (Shenzhen Pingshan Traditional Chinese Medicine Hospital), Shenzhen, China

**Keywords:** amyotrophic lateral sclerosis, degenerative cervical myelopathy, differential diagnosis, electrodiagnosis, explanatory sufficiency, magnetic resonance imaging

## Abstract

Differentiating amyotrophic lateral sclerosis (ALS) from degenerative cervical myelopathy (DCM) remains difficult because the two disorders can converge clinically while diverging biologically. ALS is a progressive motor neuron disease, whereas DCM is a potentially treatable compressive myelopathy; however, both may present with upper-limb weakness, hand wasting, hyperreflexia, gait disturbance, and cervical MRI abnormalities. This narrative review examines ALS-DCM overlap through the concept of explanatory sufficiency: whether the available clinical, imaging, and electrophysiological evidence adequately explains the whole syndrome rather than a single visible abnormality. We synthesize evidence on phenotype-specific overlap, MRI-clinical mismatch, EMG/NCS distribution, somatosensory and motor evoked potentials, Gold Coast diagnostic criteria, primary lateral sclerosis, and coexistence of motor neuron disease with structural cervical pathology. The review emphasizes that MRI is indispensable but not self-interpreting, EMG/NCS is most useful when interpreted by distribution rather than positivity alone, and SEPs/MEPs can add a functional cord-conduction layer when MRI and examination are discordant. We also provide action-oriented clinical warning signs for common overlap scenarios. Rather than offering a rigid algorithm, this review proposes a clinically driven reasoning framework that helps distinguish ALS, DCM, radiculopathy, and coexistence while reducing premature diagnostic closure in neuro-spine practice.

## 1 Introduction

ALS and DCM occupy different biological territories, yet they repeatedly collide in the clinic. ALS is defined by progressive degeneration of upper and lower motor neurons and is increasingly understood as a heterogeneous clinicopathological continuum rather than a single uniform entity (1–4). DCM, by contrast, reflects chronic degenerative narrowing of the cervical canal with mechanical, ischemic, inflammatory, and neurophysiological injury to the spinal cord (5–11). This distinction is straightforward in textbooks but less clean in practice. A patient with hand wasting, brisk reflexes, gait slowing, and cervical stenosis may have DCM, ALS with incidental spondylosis, or both. The diagnostic task is therefore not simply to choose one disease label, but to determine whether the observed abnormalities are sufficient to explain the whole evolving syndrome.

The stakes are high. DCM is the most common cause of non-traumatic spinal cord dysfunction in adults and is often underrecognized until disability has accumulated (5–9). Timely recognition matters because appropriate surgical decompression can prevent further neurological decline in selected patients (6, 12, 13). ALS, meanwhile, remains incurable and frequently experiences diagnostic delay, partly because early symptoms can resemble focal orthopedic or radicular disease (14–17). Mislabeling ALS as cervical myelopathy may lead to unnecessary decompression and delayed neurologic care; missing DCM may withhold a potentially disease-modifying intervention. This is why the ALS-DCM boundary is not a marginal academic issue but a recurring site of clinical risk in spine, neurology, rehabilitation, and primary care.

The literature has improved substantially in both fields, but it has not fully solved the overlap problem. DCM reviews and guidelines have clarified symptom clusters, imaging metrics, natural history, and operative indications (6–13, 18–23). ALS diagnostic work has moved from El Escorial and Awaji-era thinking toward the Gold Coast approach, which increases diagnostic sensitivity while retaining the importance of progression and exclusion of mimics (24–32). Separate progress in each disease, however, can paradoxically sharpen rather than resolve the problem at the interface. High-resolution MRI detects many degenerative abnormalities, including in asymptomatic individuals (33–35), while modern ALS criteria allow earlier recognition of regional motor neuron disease (24, 28–31). The clinician must therefore ask a more difficult question: which finding is explanatory, which is incidental, and which is still immature because the disease has not yet declared itself?

Most previous discussions frame DCM as one item in the list of ALS mimics, or ALS as one neurological mimic of DCM (14, 36–39). That approach is useful but incomplete. Diagnostic confusion rarely occurs between a fully typical ALS case and a fully typical myelopathic DCM case. It usually appears in phenotype-specific border zones: flail arm syndrome with cervical stenosis, lower motor neuron-predominant ALS with foraminal narrowing, primary lateral sclerosis (PLS) with mild cord compression, or myelopathic DCM with focal wasting. A more clinically faithful review therefore needs to move from disease-to-disease comparison toward phenotype-to-phenotype reasoning.

**Figure 1:**
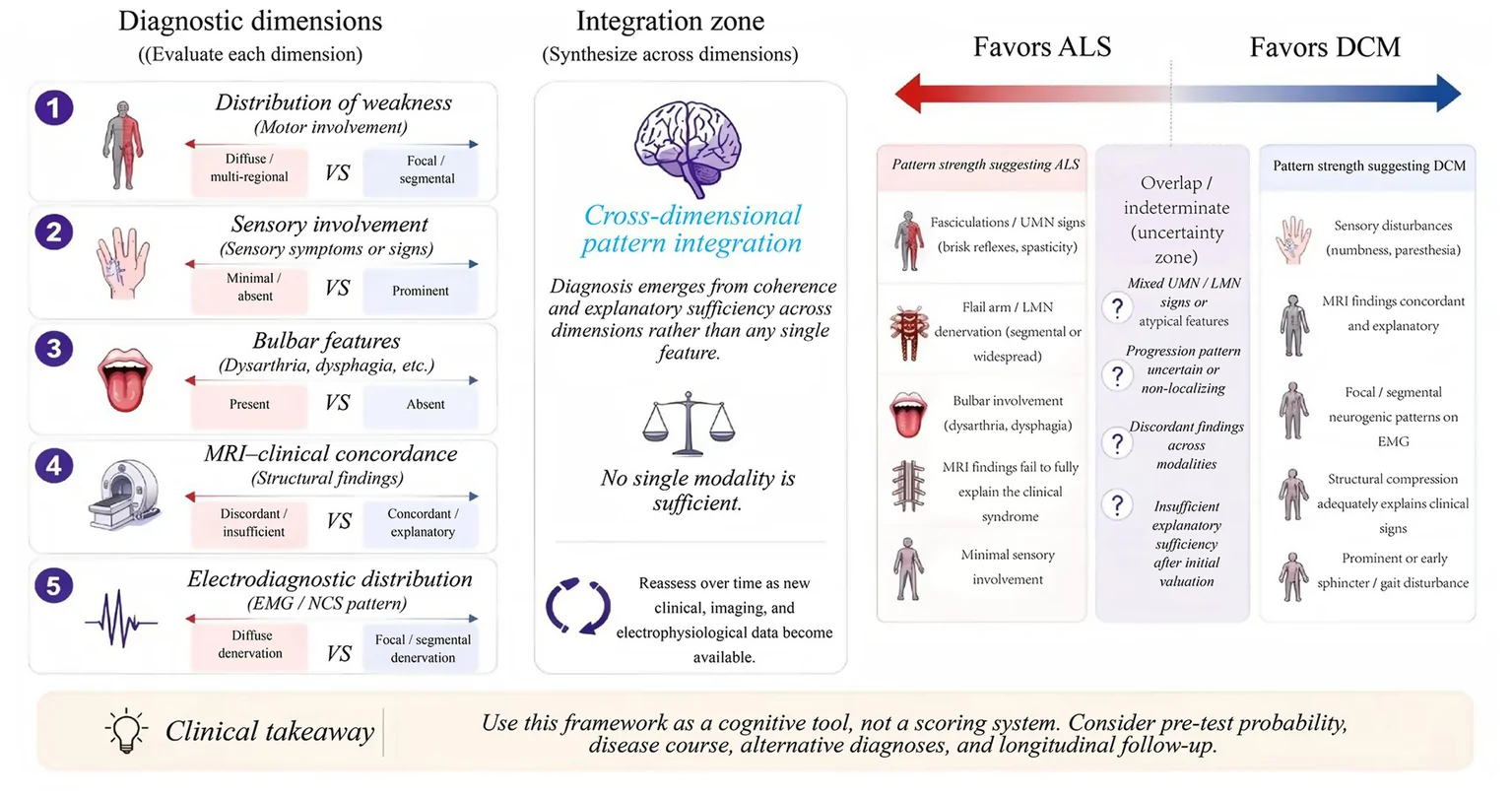
Conceptual overlap between ALS and DCM. The figure summarizes why ALS and DCM intersect clinically despite distinct mechanisms. ALS reflects progressive motor neuron degeneration, whereas DCM reflects compressive cervical cord and root pathology. The central diagnostic challenge occurs in the overlap zone, where upper-limb weakness, hand wasting, hyperreflexia, gait disturbance, and cervical MRI abnormalities may coexist. Differentiation requires explanatory sufficiency rather than reliance on a single sign, test, or imaging abnormality.

This review develops such an approach. After a brief description of the literature selection principles, we examine why ALS-DCM differentiation is intrinsically difficult, map the main phenotype-specific overlap zones, and then analyze the diagnostic value and limitations of examination, MRI, EMG/NCS, SEPs, MEPs, and longitudinal reassessment. Figure 1 summarizes the conceptual problem: ALS, DCM, and their overlap are best interpreted not by disease labels alone, but by whether the clinical syndrome is sufficiently explained by the available evidence.

## 2 Literature search and selection principles

This narrative review was based on targeted searches of PubMed/MEDLINE, Web of Science, Embase, and Google Scholar, supplemented by reference-chain screening of key reviews, diagnostic criteria papers, guidelines, and clinically instructive case reports. Priority was given to literature addressing ALS phenotypes, DCM recognition, neurological mimics, MRI-clinical concordance, EMG/NCS, SEPs/MEPs, Gold Coast criteria, PLS, and misdiagnosis at the neuro-spine interface. We included recent evidence where available while retaining landmark articles that remain central to diagnostic reasoning, including classic ALS criteria, DCM guidelines, and studies of asymptomatic cervical degeneration.

## 3 Why ALS-DCM differentiation is difficult

### 3.1 Anatomical and pathological convergence

ALS and DCM become difficult to separate because their clinical signs converge at the cervical cord and anterior horn interface. Cervical cord compression can produce hand clumsiness, weakness, wasting, hyperreflexia, spastic gait, proprioceptive disturbance, and sphincter symptoms; ALS can produce a similar mixture of upper and lower motor neuron signs when onset is cervical or upper-limb dominant (1–4, 10–13, 18, 19). A patient with hand wasting, brisk reflexes, gait slowing, and cervical stenosis could have DCM, ALS with incidental spondylosis—or both. The diagnostic task is therefore not to decide whether an abnormality exists, but whether it has sufficient explanatory power for the whole syndrome.

Mechanistically, DCM and ALS are not interchangeable. DCM begins with chronic cord deformation, ischemia, blood-spinal cord barrier disruption, demyelination, neuroinflammation, and secondary axonal loss (10, 11). ALS reflects a multisystem neurodegenerative process involving motor neurons, glia, corticospinal tracts, and extra-motor networks (1–4). Yet these distinct biological routes can terminate in overlapping motor phenotypes. Sensory symptoms can help, but they are not an absolute divider: prominent dermatomal sensory loss or proprioceptive impairment supports DCM, whereas relatively preserved sensation in a progressive motor syndrome should keep ALS in the differential (23, 37, 40).

### 3.2 Imaging visibility and the risk of over-attribution

The second difficulty is radiological abundance. MRI is indispensable for detecting cervical compression. However, it is vulnerable to overinterpretation because asymptomatic degenerative changes are common. The systematic review by Brinjikji et al. (34) and the classic cervical MRI study by Boden et al. (35) remain cautionary examples: visible degeneration is frequent even in individuals without corresponding symptoms. In older patients, a cervical abnormality may therefore be clinically causal, partially explanatory, or incidental. The distinction depends on concordance with neurological level, laterality, severity, sensory findings, electrophysiology, and progression.

This point is particularly important in spine clinics, where imaging is often the most concrete piece of evidence available. Severe compression with cord signal change and a matching myelopathic syndrome has high explanatory sufficiency. Mild multilevel stenosis in a patient with sensory-sparing progressive weakness, fasciculations, bulbar symptoms, or extra-cervical denervation does not. MRI should therefore be treated as structural context rather than diagnostic closure.

### 3.3 Diagnostic criteria, phenotypic heterogeneity, and cognitive bias

The third difficulty comes from ALS heterogeneity and evolving diagnostic criteria. The Gold Coast criteria simplified ALS diagnosis and improved sensitivity, but they also place greater responsibility on the clinician to exclude structural mimics and interpret progression correctly (24–32). This is not a formality. In a patient with focal weakness and cervical stenosis, applying ALS criteria without testing MRI-clinical concordance and EMG distribution risks false certainty; conversely, attributing all weakness to stenosis delays recognition of motor neuron disease.

The final difficulty is cognitive. Once a plausible cervical lesion is seen, reasoning may anchor to that lesion and discount contradictory features. Conversely, once an ALS label is suspected, treatable DCM may be dismissed as incidental. Case reports of ALS recognized after cervical decompression, and reports of unnecessary spine surgery in neurological mimics, are weak epidemiological evidence but strong clinical warnings (17, 39, 41, 42). They show why explanatory sufficiency must be reassessed over time rather than assumed at first presentation.

## 4 Phenotype-specific overlap patterns

### 4.1 Classical spinal-onset ALS versus classical myelopathic DCM

Classical spinal-onset ALS and classical myelopathic DCM overlap through the combination of limb weakness, brisk reflexes, gait disturbance, and hand dysfunction. In ALS, weakness often begins focally and then spreads regionally or contiguously, with relatively preserved sensation and eventual involvement outside a single spinal level (1, 2, 43–45). In DCM, the syndrome is more likely to remain anatomically interpretable from the cervical lesion: hand clumsiness, sensory disturbance, proprioceptive impairment, gait imbalance, and sphincter symptoms may cluster around a compressive myelopathy (5–9, 46–50).

The distinction becomes clinically meaningful when MRI only partly explains the syndrome. A severe C4-C5 compression with cord signal change, bilateral hand dysfunction, long-tract signs, sensory symptoms, and gait impairment is different from mild multilevel spondylosis in a patient with progressive asymmetric wasting and no sensory correlate. The first pattern supports DCM; the second should keep ALS high in the differential even if degenerative changes are visible. The literature on misdiagnosis and unnecessary spine surgery illustrates this problem: structural abnormalities can be real yet non-explanatory (17, 39, 41, 42).

### 4.2 Flail arm syndrome versus upper-limb-dominant DCM

Flail arm syndrome is one of the most treacherous border zones because it may appear regionally cervical for a prolonged period. Wijesekera et al. (51) described the natural history of flail arm and flail leg ALS variants, emphasizing distinctive regional phenotypes with slower progression than classical ALS. Hubers et al. further characterized flail arm syndrome as a phenotype dominated by upper-limb weakness and wasting, often requiring careful separation from structural cervical disease (52). A later study focusing on UMN signs in the cervical region of flail arm syndrome also showed why the label cannot rest on wasting alone (53).

Upper-limb-dominant DCM can mimic this presentation when compression affects segments related to upper-extremity function. Cervical spondylotic amyotrophy and radiculopathy-dominant degenerative disease may present with weakness and atrophy before gait symptoms are prominent (18, 36). The practical discriminator is not simply proximal versus distal weakness. It is whether the deficit remains segmentally plausible, whether pain and sensory changes are meaningful rather than incidental, whether MRI compression corresponds to the deficit, and whether EMG abnormalities remain restricted. Progressive loss of anatomical confinement, diffuse fasciculations, and denervation beyond expected roots should shift the interpretation toward motor neuron disease (26, 44, 52–54).

Cervical spondylotic amyotrophy deserves separate consideration because it may present as motor-predominant upper-limb wasting with limited sensory symptoms, thereby resembling LMN-predominant ALS or PMA. However, cervical spondylotic amyotrophy usually remains anatomically segmental, often corresponds to anterior horn or root-level cervical pathology, and lacks bulbar involvement or progressive extra-cervical spread. In contrast, LMN-predominant ALS/PMA becomes more likely when weakness and denervation extend beyond a single myotomal or root distribution (18, 26, 36, 44, 52–54).

### 4.3 LMN-predominant ALS or PMA versus cervical radiculopathy

LMN-predominant ALS and PMA-spectrum disease are especially vulnerable to misclassification because UMN signs may be absent or clinically subtle early. Van den Berg-Vos et al. demonstrated that adult-onset sporadic lower motor neuron disease comprises heterogeneous subtypes rather than a single benign category (54). In the cervical region, radiculopathy can also produce focal weakness, depressed reflexes, and wasting. If pain and sensory disturbance follow a dermatomal pattern and MRI shows concordant root compression, a radicular explanation becomes persuasive. If weakness spreads across multiple non-contiguous myotomes, sensation is preserved, fasciculations are widespread, and EMG shows active and chronic denervation beyond one root, the radicular model becomes insufficient (26, 27, 54–59).

This scenario is where EMG/NCS has its greatest added value. The result is not merely ‘positive’ or ‘negative’. The distribution matters: paraspinal and limb muscles, cervical versus lumbosacral regions, bulbar or thoracic sampling, active denervation, chronic reinnervation, sensory nerve action potentials, and conduction block all influence interpretation (26, 27, 55–59). A cervical MRI cannot adjudicate a multiregional motor neuron process by itself.

### 4.4 UMN-predominant ALS, PLS, and spastic gait-dominant DCM

The overlap between UMN-predominant motor neuron disease and spastic gait-dominant DCM is conceptually different from the LMN problem. Here, the difficulty is not wasting but the interpretation of spasticity, hyperreflexia, and gait impairment. DCM commonly produces gait dysfunction through corticospinal and posterior column involvement, and quantitative gait studies have helped objectify this clinical domain (46). UMN-predominant ALS may also present with progressive spasticity. PLS must be handled separately: the 2020 consensus criteria require a sustained pure UMN syndrome and exclusion of significant LMN involvement, with the temporal threshold central to diagnostic confidence (60, 61).

The clinical trap is to compress these entities into a single category of ‘UMN disease’. A patient with spastic paraparesis, sensory loss, hand numbness, urinary urgency, and severe cervical compression has a different probability structure from a patient with slowly progressive bilateral spasticity, normal sensation, limited MRI explanation, and later bulbar features. The former supports DCM; the latter demands sustained neurological follow-up and repeated electrophysiological assessment for PLS or UMN-predominant ALS (60–63).

**Figure 2:**
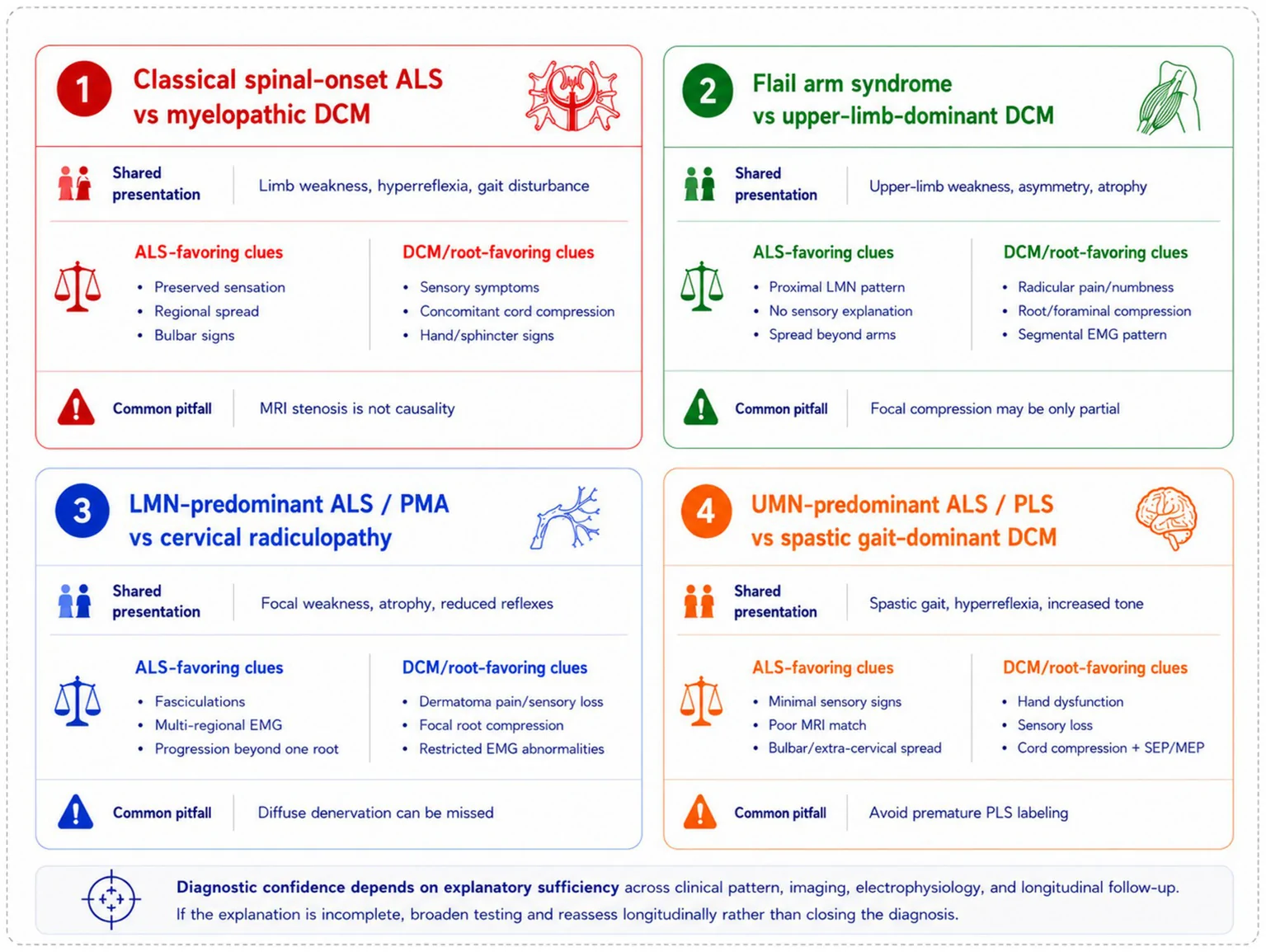
Phenotype-specific overlap zones and key discriminators. This multi-panel schematic summarizes four common overlap scenarios in ALS-DCM differentiation: Classical spinal-onset ALS versus myelopathic DCM, flail arm syndrome versus upper-limb-dominant DCM, LMN-predominant ALS/PMA versus cervical radiculopathy, and UMN-predominant ALS/PLS versus spastic gait-dominant DCM. Each panel highlights shared presentations, ALS-favoring clues, DCM/root-favoring clues, and common diagnostic pitfalls. The figure emphasizes that overlap reflects pattern similarity rather than shared mechanisms, and that diagnostic confidence depends on explanatory sufficiency across clinical pattern, imaging, electrophysiology, and longitudinal follow-up.

These four overlap zones are summarized in Figure 2 and Table 1. They should not be read as separate diagnostic boxes: a patient can move between them over time, and more than one mechanism may be present. The next section therefore asks which diagnostic dimensions—clinical examination, MRI, EMG/NCS, SEPs/MEPs, and longitudinal evolution—most strongly increase or weaken explanatory sufficiency within these overlap scenarios.

**Table 1:** Key phenotype-specific overlap scenarios.

Overlap scenario	Why it is confusing	ALS-spectrum clues	DCM/radicular clues	Highest-yield evaluation
Spinal-onset ALS vs. myelopathic DCM	Weakness, hyperreflexia, gait decline, and hand dysfunction overlap	Motor spread; fasciculations; bulbar or respiratory involvement	Sensory/proprioceptive loss; sphincter symptoms; concordant cord compression	Examination + MRI concordance; EMG/NCS if mismatch persists
Flail arm syndrome vs. upper-limb-dominant DCM	Both may begin with arm weakness and wasting	Loss of segmental confinement; minimal sensory explanation	Radicular pain/numbness; segmentally plausible compression	Serial examination; EMG beyond the symptomatic limb
LMN-predominant ALS/PMA vs. cervical radiculopathy	Both may show focal wasting and reduced reflexes	Diffuse active/chronic denervation; preserved sensory responses	Dermatomal symptoms; focal root-level EMG; foraminal lesion	EMG/NCS distribution and repeat assessment
UMN-predominant ALS/PLS vs. spastic gait-dominant DCM	Both may present with spasticity and gait impairment	Poor MRI-clinical concordance; later bulbar or extra-cervical signs	Cord compression matching pyramidal signs; posterior-column symptoms	MRI-clinical concordance; SEP/MEP; longitudinal EMG

## 5 Diagnostic dimensions: from findings to explanatory sufficiency

### 5.1 Clinical examination remains the first line of assessment

The DCM literature consistently emphasizes that early recognition depends on history and examination, not MRI alone (7, 23, 47–50, 64). Hand clumsiness, gait imbalance, sensory disturbance, hyperreflexia, pathological reflexes, and sphincter symptoms are clinically meaningful because they define the syndrome that imaging must explain. In ALS-DCM overlap, the same principle applies with higher stakes: the examination is the first line of assessment because it determines whether a cervical explanation is anatomically plausible.

For ALS-DCM differentiation, the examination findings should be interpreted in a spatial context. Does weakness respect a root, cord level, or peripheral nerve? Are reflexes reduced only in muscles supplied by compressed roots, or are they pathologically brisk in remote territories? Are sensory deficits dermatomal, posterior-column predominant, or absent despite progressive motor decline? The explanatory sufficiency of the initial diagnosis increases when these findings fit together; it weakens when the examination describes a syndrome larger than the visible lesion.

### 5.2 MRI: indispensable, but not self-interpreting

MRI is indispensable for DCM because it identifies canal stenosis, cord compression, T2 hyperintensity, T1 hypointensity, dynamic or multilevel compression, and alternative structural diagnoses (20–22, 33). However, MRI is not self-interpreting. A visible lesion becomes diagnostically meaningful only when it explains the clinical level, laterality, severity, sensory pattern, and progression of the syndrome.

The strongest MRI argument for DCM is concordance. Severe compression with matching hand dysfunction, long-tract signs, proprioceptive loss, gait impairment, and sphincter symptoms has high explanatory sufficiency. In contrast, mild or multilevel degenerative change in a patient with sensory-sparing progressive weakness, fasciculations, bulbar symptoms, or denervation beyond the cervical region should be interpreted cautiously (17, 34–36, 39, 41, 42).

**Figure 3:**
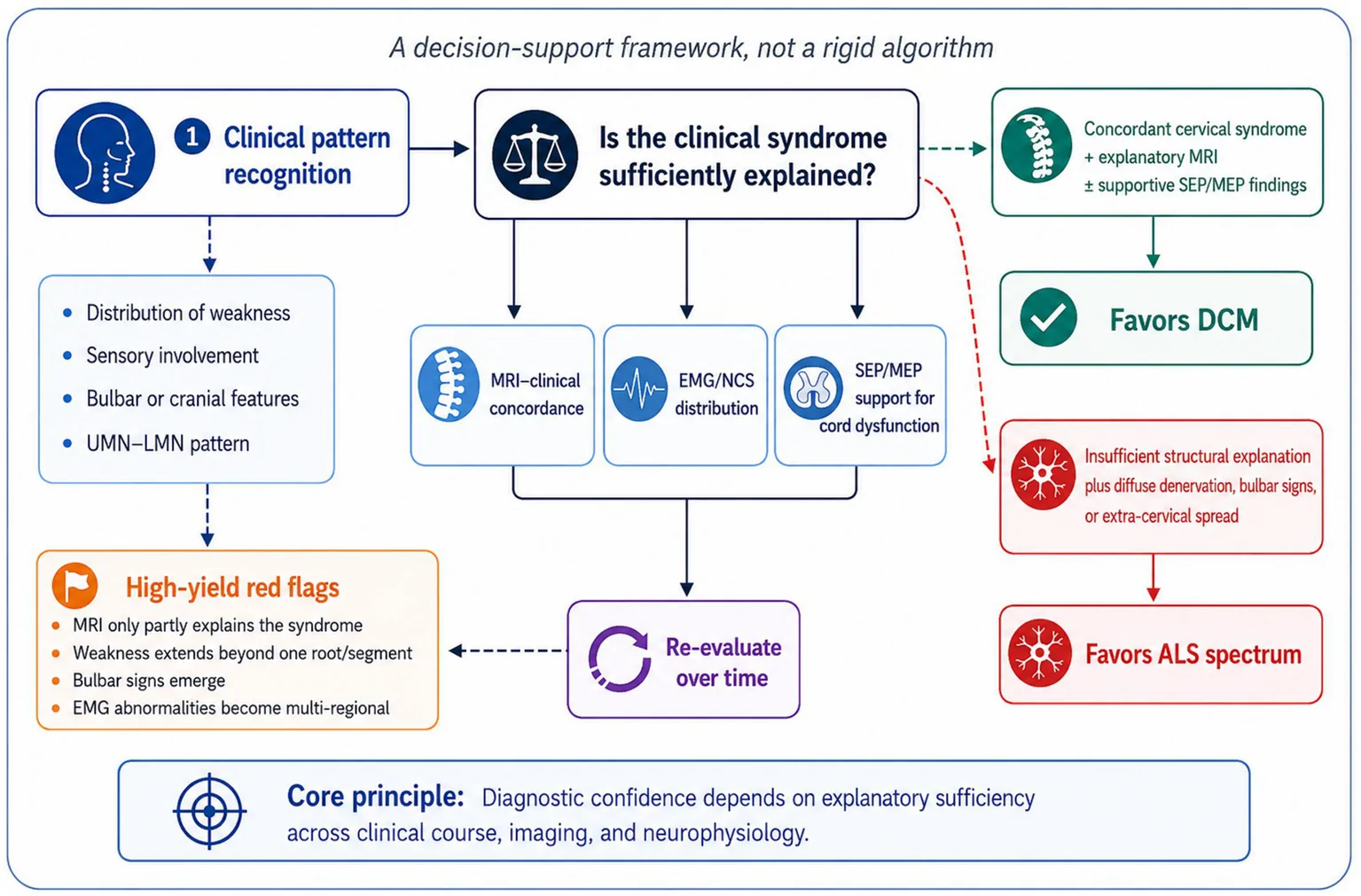
Clinically driven framework for ALS-DCM differentiation. This figure illustrates a phenotype-first decision-support framework for evaluating ALS-DCM overlap. The central step is determining whether the clinical syndrome is sufficiently explained by one coherent anatomical diagnosis. MRI-clinical concordance, EMG/NCS distribution, and SEP/MEP evidence of cord dysfunction are interpreted as complementary dimensions rather than as a fixed sequence of tests. Concordant cervical syndromes favor DCM, whereas insufficient structural explanation combined with diffuse denervation, bulbar signs, or extra-cervical spread favors ALS spectrum disease. Longitudinal reassessment is emphasized when explanatory sufficiency remains incomplete.

At this point, the central diagnostic question is not whether cervical MRI is abnormal, but whether the visible lesion sufficiently explains the entire clinical syndrome. Figure 3 operationalizes this principle by separating concordant cervical syndromes from cases in which structural findings are only partially explanatory, thereby reducing the risk that MRI visibility is mistaken for MRI causality.

### 5.3 EMG/NCS: resolving distributional ambiguity

EMG/NCS is most useful when the clinical and imaging narratives do not align. ALS criteria and electrodiagnostic literature emphasize that LMN dysfunction can be established clinically or by EMG, and that the distribution of active and chronic denervation is central to interpretation (24–32, 55–58). In the spine setting, EMG should not be reduced to a binary test. Its value lies in whether abnormalities remain anatomically restricted or extend beyond the structural explanation.

Diffuse active and chronic denervation across multiple regions, especially with preserved sensory nerve action potentials and abnormalities outside MRI-defined compression, supports ALS-spectrum disease. Abnormalities confined to a single root distribution, with radicular pain or sensory loss and concordant foraminal stenosis, support radiculopathy or DCM-related root involvement. EMG/NCS increases explanatory sufficiency when its spatial pattern matches the proposed diagnosis; it weakens that diagnosis when it reveals a process too broad for the visible lesion.

### 5.4 SEPs and MEPs: the often-underused cord-function layer

SEPs and MEPs deserve more explicit attention in ALS-DCM overlap. EMG is excellent for detecting LMN involvement, but it does not directly quantify dorsal column or corticospinal conduction across a compressed cervical cord. SEPs can reveal delayed, low-amplitude, or absent cortical responses after peripheral stimulation, including N20 abnormalities after median nerve stimulation when the posterior column-medial lemniscal pathway is functionally impaired. MEPs can demonstrate corticospinal dysfunction through prolonged central motor conduction time, reduced motor responses, or absent responses when interpreted against laboratory norms (55, 59, 65).

In practical terms, SEPs/MEPs are most useful when the structural and clinical evidence is discordant. If MRI shows only moderate stenosis but examination strongly suggests myelopathy, abnormal SEPs or prolonged CMCT can strengthen confidence that the cord lesion is functionally relevant. Conversely, if a patient has a pure motor syndrome, preserved sensation, insufficient MRI concordance, and no evoked-potential evidence of cord conduction failure, the explanatory balance shifts toward ALS-spectrum disease and broader EMG sampling becomes more important.

These tests should not replace EMG/NCS or clinical examination. Their role is complementary: they add a functional cord-conduction layer to the question of explanatory sufficiency. This is particularly valuable when surgical decision-making depends on whether cervical compression is merely visible or truly responsible for disability.

The complementary diagnostic contributions and limitations of clinical examination, MRI, EMG/NCS, SEPs/MEPs, and longitudinal reassessment are summarized in Table 2.

**Table 2:** Concise diagnostic dimensions in ALS-DCM overlap.

Dimension	Clues favoring DCM/radiculopathy	Clues favoring ALS-spectrum disease	Practical emphasis
Weakness pattern	Segmental or root-level distribution; anatomically coherent signs	Spread beyond one root/segment or loss of anatomical confinement	Map distribution and progression before assigning causality
Sensory and pain features	Dermatomal pain, numbness, proprioceptive loss, or sphincter symptoms	Progressive motor decline with relatively preserved sensation	Use sensory findings as explanatory evidence, not labels
Bulbar or extra-cervical signs	Not explained by focal cervical disease	Dysarthria, dysphagia, tongue fasciculations, respiratory decline, or remote spread	Reopen the diagnosis when these features appear
MRI-clinical relationship	Compression matches level, laterality, severity, and syndrome	Stenosis explains only part of the syndrome	Treat MRI as a hypothesis, not a final diagnosis
EMG/NCS pattern	Focal root/segment abnormalities concordant with imaging	Multi-region denervation or abnormalities beyond the cervical territory	Sample beyond the symptomatic limb when mismatch persists
SEP/MEP and follow-up	Cord conduction abnormality supports functional myelopathy	Normal cord conduction or evolving extra-cervical signs weakens a purely cervical explanation	Reassess longitudinally if explanatory sufficiency is incomplete

### 5.5 Bulbar, respiratory, cognitive, and extra-cervical features

Bulbar involvement remains a powerful counterweight to cervical anchoring. Dysarthria, dysphagia, tongue atrophy, tongue fasciculations, and respiratory decline are not explained by focal cervical stenosis. Their emergence should trigger reconsideration even if cervical imaging appears convincing. ALS is also increasingly recognized as part of a broader neurodegenerative spectrum with cognitive and behavioral involvement in a subset of patients (1–4, 66). These features do not replace motor examination, but they change the diagnostic probability landscape.

### 5.6 Motor-pattern clues: useful but never decisive alone

Phenotypic motor-pattern clues can sharpen diagnostic reasoning when they are interpreted cautiously. The split-hand phenomenon, for example, has been repeatedly discussed as a characteristic ALS pattern in which thenar and first dorsal interosseous involvement is disproportionately greater than hypothenar involvement (67, 68). In an upper-limb presentation with limited sensory symptoms, such a pattern increases suspicion for ALS-spectrum disease. However, it should not be treated as pathognomonic. Cervical radiculopathy, entrapment neuropathy, prior trauma, and disuse can distort hand muscle bulk and strength. The correct use of split-hand or split-limb observations is therefore probabilistic: they help determine where to sample on EMG and whether the structural lesion is sufficient, but they should not replace anatomical localization.

This nuance is important because high-level reviews sometimes translate group-level observations into bedside shortcuts. A rigorous interpretation should avoid that shortcut. ALS and DCM are both clinically heterogeneous, and a sign that is useful in cohort discrimination may be weaker in a single patient with coexisting stenosis, diabetes, peripheral neuropathy, or prior cervical surgery. The strongest diagnostic argument is a constellation: motor distribution, sensory profile, reflex dissociation, MRI concordance, EMG extent, evoked-potential evidence, and longitudinal evolution pointing in the same direction.

## 6 A clinically driven diagnostic framework

A useful diagnostic framework begins with the clinical syndrome, not with MRI. The first step is to name the phenotype: classical myelopathic, radicular, flail arm, LMN-predominant, UMN-predominant, PLS-like, or mixed. The second step is to ask whether the proposed diagnosis explains the patient across domains: motor distribution, sensory findings, reflexes, bulbar screen, MRI concordance, EMG/NCS distribution, evoked potentials, and time course.

Figure 3 translates this reasoning into a flexible decision-support framework rather than a rigid sequence. Radicular pain, dermatomal sensory loss, and concordant cord or root compression support early structural evaluation. In contrast, sensory-sparing progressive weakness, fasciculations, bulbar symptoms, extra-cervical spread, or multi-regional denervation should prompt early EMG/NCS, consideration of SEPs/MEPs when cord dysfunction is uncertain, and longitudinal reassessment when explanatory sufficiency remains incomplete.

When clinical, imaging, and electrophysiological evidence converge, explanatory sufficiency increases and the diagnostic pathway becomes more stable. When only one modality explains the syndrome, the safest response is not forced classification but re-evaluation. This is the practical meaning of explanatory sufficiency: diagnostic confidence should be proportional to how completely the available evidence accounts for the patient, not to how persuasive one isolated finding appears.

### 6.1 Red flags and action-oriented clues

Because the most useful diagnostic clues are scattered across examination, imaging, and electrophysiology, Table 3 summarizes practical warning signs and suggested actions. The table is not intended to replace clinical judgment. Its purpose is to make the framework usable at the bedside: identify when a cervical explanation is strong, when ALS should remain high on the differential, and when coexistence or re-evaluation is the most defensible interpretation.

**Table 3:** Action-oriented red flags in ALS-DCM overlap.

Clinical clue	Main concern	Suggested action
MRI only partly explains the syndrome	Incidental stenosis or coexistence	Reassess; expand EMG/NCS sampling
Weakness extends beyond one root/segment	Motor neuron spread	Examine other regions and repeat EMG if needed
Bulbar or respiratory signs emerge	ALS-spectrum disease	Prioritize neurological review
EMG abnormalities become multi-regional	Diffuse motor neuron involvement	Apply ALS criteria while excluding mimics
Concordant compression with sensory, gait, or urinary signs	Clinically meaningful DCM	Consider spine referral; SEP/MEP may support cord dysfunction

### 6.2 Coexistence: when both explanations are partly true

Coexistence is not a diagnostic paradox. An older patient may have ALS and clinically meaningful DCM, or ALS with incidental stenosis, or DCM with unrelated peripheral neuropathy. The presence of ALS does not prove that cervical compression is irrelevant; equally, decompression cannot be expected to reverse a diffuse motor neuron process. Robles and Chakravarthy illustrated this dilemma in a patient with motor neuron disease and cervical spondylotic myelopathy, emphasizing that simultaneous explanations can coexist without sharing causality (41). In practice, surgery should be considered only when the DCM component has its own explanatory sufficiency: severe or progressive compression, concordant long-tract signs, sensory or sphincter features, and supportive functional evidence. In mixed cases, the realistic goal may be stabilization or relief of compressive symptoms, not reversal of ALS-driven weakness.

## 7 Limitations of current evidence

The evidence base is clinically useful but uneven. DCM has strong guideline literature and increasing work on diagnostic criteria, natural history, and clinical recognition (6–13, 18–22, 46–50, 64, 69, 70). ALS evidence has advanced through diagnostic criteria, biomarker development, neurophysiology, and phenotype studies (24–32, 71–74). Yet the most clinically difficult population—patients in whom ALS and DCM are both plausible—remains under-studied. Much of the available evidence comes from retrospective cohorts, mimic reviews, case reports, surgical referral series, and modality-specific studies rather than prospective overlap registries.

The Gold Coast criteria illustrate both progress and risk. Their simplified structure improves diagnostic sensitivity and reduces dependence on older category labels, but in spine clinics they must be applied with particular caution (24, 27–32). Progression and exclusion of structural mimics are not administrative requirements; they are the safeguards that prevent a focal compressive syndrome from being mislabeled as ALS. In patients with cervical stenosis and focal weakness, Gold Coast criteria should be applied only after careful assessment of MRI-clinical concordance and electrodiagnostic distribution.

A second limitation is terminology. Terms such as ALS mimic, DCM mimic, incidental stenosis, and coexisting disease imply different causal models but are often used loosely. A mimic is a false diagnostic substitute; coexistence means two true processes; incidental imaging means a visible lesion without adequate explanatory power. Without clearer terminology, studies may appear to address the same problem while actually describing different diagnostic situations.

Several biases also shape the literature. Surgical DCM cohorts are enriched for patients with convincing compression, whereas neuromuscular referral cohorts are enriched for atypical motor syndromes. Case reports highlight memorable failures but cannot estimate frequency. Advanced MRI, neurofilament biomarkers, and quantitative neurophysiology may improve future differentiation, but their value should be tested in the grey-zone patients who create real diagnostic difficulty, not only in typical ALS versus healthy controls (40, 66, 71–80).

Future studies should therefore compare diagnostic strategies, not merely diagnostic entities. Reports should state weakness distribution, sensory examination, reflex pattern, bulbar screen, MRI level and severity, EMG sampling strategy, SEP/MEP findings, treatment decisions, and longitudinal outcome. The goal should not be a simplistic score, but a validated estimate of explanatory sufficiency: how strongly each modality accounts for the patient at that moment, and when reassessment is required.

## 8 Practical implications for neuro-spine practice

For spine surgeons, the central lesson is that decompression decisions should not be based on imaging severity alone when motor-neuron features are atypical. Progressive sensory-sparing weakness, diffuse fasciculations, bulbar signs, or EMG abnormalities beyond the compressed level should trigger neurological evaluation before surgery. When DCM is still present and clinically explanatory, surgical discussion can remain appropriate, but expected benefit should be framed around the compressive component rather than the total neurological burden.

For neurologists, the mirror-image lesson is equally important. Cervical compression is not always incidental, and ALS criteria do not remove the obligation to identify treatable myelopathy. Neck pain, dermatomal sensory loss, proprioceptive impairment, urinary urgency, concordant cord compression, and abnormal SEPs/MEPs should prompt careful spine assessment even when ALS is suspected.

For rehabilitation and primary care clinicians, the key is early pattern recognition. Worsening hand function, gait instability, falls, unexplained upper-limb wasting, fasciculations, dysphagia, and imaging-clinical mismatch should not be managed as isolated musculoskeletal complaints. Timely referral is most likely when clinicians ask a simple question: does one diagnosis truly explain the whole syndrome, or is explanatory sufficiency incomplete?

## 9 Research agenda: validating explanatory sufficiency

Future research should move beyond retrospective lists of mimics toward prospective neuro-spine overlap registries. A minimum dataset should include standardized weakness distribution, sensory mapping, reflex profile, gait measures, bulbar screening, MRI severity and concordance grading, EMG/NCS distribution, SEPs/MEPs, surgical decision-making, and longitudinal diagnostic outcome. Such studies would allow the field to test whether explanatory sufficiency can be operationalized rather than left as an implicit clinical instinct.

Advanced MRI, diffusion metrics, neurofilament biomarkers, and quantitative neurophysiology may refine this interface (40, 66, 71–80). Their role, however, should be judged critically. A tool that separates typical ALS from healthy controls may not solve the harder problem of ALS versus DCM with overlapping signs. Translational value will depend on whether these tools change management in the exact diagnostic grey zones where clinicians hesitate.

## 10 Conclusion

ALS-DCM differentiation is best understood as a problem of explanatory sufficiency within phenotype-specific overlap scenarios. The clinician must decide whether a cervical lesion explains the distribution, severity, sensory features, electrophysiology, evoked potentials, and evolution of the syndrome, or whether a motor neuron process better accounts for the whole pattern. MRI, EMG/NCS, SEPs, MEPs, and clinical examination each answer different questions; none is sufficient alone. A visible cervical abnormality should not end diagnostic reasoning, and an ALS label should not automatically erase treatable cervical disease. By shifting attention from isolated findings to the coherence of the whole syndrome, this review offers a practical framework for reducing premature closure, recognizing coexistence, and guiding more defensible decisions in neuro-spine practice.
