# Supplementary material for: Amyotrophic lateral sclerosis and degenerative cervical myelopathy: phenotype-based diagnostic pitfalls, investigative mismatch, and practical clinical reasoning
Source: Front Neurol. 2026 Aug 6;17:1901811. doi: 10.3389/fneur.2026.1901811 (PMC13490098; doi:10.3389/fneur.2026.1901811)
Supplement: Supplementary file 1 [file Data_Sheet_1.DOCX]

**Supplementary File S1. Generative AI Prompts and Outputs for Figure Optimization**

Manuscript: Amyotrophic Lateral Sclerosis and Degenerative Cervical Myelopathy: Phenotype-Based Diagnostic Pitfalls, Investigative Mismatch, and Practical Clinical Reasoning

## 1. AI tool information and scope of use

AI tool used: ChatGPT 5.2 Plus (OpenAI).

Purpose of use: AI-assisted tools were used only for optimization and visual refinement of Figures 2 and 3. They were used to improve visual organization, layout consistency, readability, and publication-quality presentation. The AI-assisted process did not involve generating scientific concepts, modifying the diagnostic framework, interpreting literature, analyzing data, or writing the manuscript.

## 2. Figure 2 optimization

Input prompt provided to the AI-assisted tool:

Please refine this medical infographic figure for a high-quality SCI review article. Maintain the original scientific framework and diagnostic logic. Improve visual hierarchy, readability, color consistency, spacing, and professional medical illustration style. Simplify unnecessary visual complexity while preserving all clinically important information. Do not alter the scientific interpretation or conclusions.

Output received from the AI-assisted tool:

The output shown above represents the AI-assisted visual refinement result. The final figure was reviewed, adjusted, and approved by the authors before manuscript submission. The scientific framework, diagnostic interpretation, and clinical content were developed by the authors.

## 3. Figure 3 optimization

Input prompt provided to the AI-assisted tool:

Please simplify and optimize this phenotype-based ALS-DCM overlap schematic for a high-impact SCI review article. Maintain the four clinical overlap scenarios and key diagnostic discriminators. Improve visual clarity, reduce excessive text, and create a clean professional medical infographic style suitable for publication. Do not modify the underlying scientific concepts.

Output received from the AI-assisted tool:

The output shown above represents the AI-assisted visual refinement result. The final figure was reviewed, adjusted, and approved by the authors before manuscript submission. The phenotype classification, clinical reasoning framework, and scientific interpretation were developed by the authors.

## 4. Author responsibility statement

The authors confirm that AI-assisted tools were used only for figure optimization and visual refinement of Figures 2 and 3. All scientific content, including the conceptual framework, literature synthesis, interpretation, and conclusions, was developed and verified by the authors. The authors take full responsibility for the accuracy, integrity, and originality of the manuscript.
